# Tools for translational neuroscience: PTSD is associated with heightened fear responses using acoustic startle but not skin conductance measures

**DOI:** 10.1002/da.20880

**Published:** 2012-09-02

**Authors:** Ebony M Glover, Justine E Phifer, Daniel F Crain, Seth D Norrholm, Michael Davis, Bekh Bradley, Kerry J Ressler, Tanja Jovanovic

**Affiliations:** 1 Department of Psychiatry and Behavioral Sciences, Emory University School of MedicineAtlanta, Georgia; 2 Yerkes National Primate Research CenterAtlanta, Georgia; 3 Atlanta VA Medical CenterDecatur, Georgia; 4 Howard Hughes Medical InstituteChevy Chase, Maryland

**Keywords:** fear-potentiated startle, skin conductance response, trauma, posttraumatic stress disorders, psychophysiology

## Abstract

*Background:* Posttraumatic stress disorder (PTSD) patients show heightened fear responses to trauma reminders and an inability to inhibit fear in the presence of safety reminders. Brain imaging studies suggest that this is in part due to amygdala over-reactivity as well as deficient top-down cortical inhibition of the amygdala. Consistent with these findings, previous studies, using fear-potentiated startle (FPS), have shown exaggerated startle and deficits in fear inhibition in PTSD participants. However, many PTSD studies using the skin conductance response (SCR) report no group differences in fear acquisition. *Method:* The study included 41 participants with PTSD and 70 without PTSD. The fear conditioning session included a reinforced conditioned stimulus (CS+, danger cue) paired with an aversive airblast, and a nonreinforced conditioned stimulus (CS−, safety cue). Acoustic startle responses and SCR were acquired during the presentation of each CS. *Results:* The results showed that fear conditioned responses were captured in both the FPS and SCR measures. Furthermore, PTSD participants had higher FPS to the danger cue and safety cue compared to trauma controls. However, SCR did not differ between groups. Finally, we found that FPS to the danger cue predicted re-experiencing symptoms, whereas FPS to the safety cue predicted hyper-arousal symptoms. However, SCR did not contribute to PTSD symptom variance. *Conclusions:* Replicating earlier studies, we showed increased FPS in PTSD participants. However, although SCR was a good measure of differential conditioning, it did not differentiate between PTSD groups. These data suggest that FPS may be a useful tool for translational research. Depression and Anxiety, 2011. © 2011 Wiley Periodicals, Inc.

## INTRODUCTION

Cardinal symptoms of posttraumatic stress disorder (PTSD) include re-experiencing a traumatic event that elicited intense feelings of fear, horror, or helplessness, increased arousal in the presence of trauma reminders, and avoidance of trauma-associated cues.^[1]^As many of the dominant features of PTSD symptoms reflect memory-related mechanisms,^[2]^ clinical research aimed at understanding fear learning processes in trauma-exposed populations continues to shed light on the biological underpinnings of the psychopathology in PTSD.

Traumatic memories may form via Pavlovian fear conditioning, whereby neutral environmental cues, or conditioned stimuli (CS), come to elicit fear- and anxiety-related behaviors, or conditioned fear responses (CR), due to their prior association with highly aversive cues, or unconditioned stimuli (US).^[3]^ Animal and human fear conditioning studies have identified the amygdala, a dense collection of neurons located deep within the temporal lobes, as a critical neuroanatomical region responsible for conditioned fear memory processing.^[4–7]^ In Pavlovian fear conditioning, sensory information about the CS and US converge in the lateral nucleus of the amygdala^[8]^ with projections to the central nucleus of the amygdala, which is the primary output nucleus of the amygdala fear circuitry. This nucleus in turn sends projections to various hypothalamic and brainstem areas, which mediate an array of fear-related physiological responses.^[6^, ^9–11]^

Although fear memories and CRs are highly adaptive mechanisms that could optimize an individual's reaction to threat, fear memories in PTSD may lead to maladaptive behaviors such as exaggerated fear responses that persist even in safe settings.^[12]^ Brain imaging studies have shown that PTSD is associated with amygdala over-reactivity as well as deficient top-down inhibition of the amygdala by the medial prefrontal cortex (mPFC) and the hippocampus.^[13–17]^ Hence, a neurobiological model is emerging that predicts dysregulated acquisition and/or inhibition of learned fear in PTSD. Studies utilizing laboratory-based fear conditioning models that probe both the expression and inhibition of learned fear in traumatized groups offer a useful approach to identify biomarkers of PTSD and uncover strategies for prevention.

In human fear conditioning models, the two most commonly measured indices of fear are an increase in skin conductance response (SCR) and acoustic startle amplitude.^[18]^ Skin conductance, which reflects changes in sweat gland activity that alters the electrical conductivity of the skin, is a direct index of sympathetic nervous system activation, and thus is an excellent measure of arousal. Importantly, the magnitude of the SCR reliably increases during presentations of a CS that was previously paired with an aversive US, making it a good index of conditioned fear.^[19–22]^ Nevertheless, the usefulness of the conditioned SCR as a phenotype of fear memory expression in PTSD research is limited by the fact that its neural correlates are not clear, in part due to a lack of animal models for this measure. Also, because a diffuse and complex neural network regulates the SCR, it may prove difficult to correlate fear-related behaviors in PTSD to specific brain centers when using this measure.

However, the startle response, an integrative motor reflex to sudden auditory stimuli, observed in all mammalian species, is mediated by a simple and short neural pathway that is directly connected to the amygdala.^[6,^^18,^^23,^^24]^ In fear-potentiated startle (FPS), the magnitude of the startle reflex increases during aversive CS presentations,^[18,^^24,^^25]^ a phenomenon that has been extensively modelled in animals.^[23,^^26]^ Hence, FPS has an advantage of being a useful translational tool that has the ability to inform the neural underpinnings of maladaptive fear in PTSD.

Our laboratory has developed a human FPS discrimination paradigm that measures startle amplitude in the presence of a reinforced conditioned stimulus (CS+)that is paired with a US, as well as during exposure to a nonreinforced conditioned stimulus (CS−) that is never paired with a US. The use of the nonreinforced CS−, which serves as a safety cue, allows us to experimentally test the idea that PTSD pathology is associated with impairments in safety signal processing.^[29–31]^ Consistent with this idea, recent findings from our group show individuals with PTSD show exaggerated FPS during both CS+(i.e. danger cue) and CS− (i.e. safety cue) presentations compared to controls.^[30,^^32]^ Other groups using FPS have found a lack of discrimination between danger and safety cues in PTSD participants due to increased responding to the CS−.^[33]^ Conversely, a number of studies using skin conductance to measure conditioned fear in PTSD versus controls did not find group differences during acquisition.^[16,^^34–36]^

The discrepancy in findings between groups that measured FPS versus skin conductance to assess fear discrimination in PTSD might be explained by the differing neurobiology of these two fear measures. Studies using arousing stimuli with a positive or negative valence suggest that the SCR is related to arousal (regardless of valence) rather than fear.^[37]^ The SCR is mediated by the lateral hypothalamus and is not a direct output of amygdala activity. FPS is more closely associated with amygdala function and may provide a more direct measure of fear. The objective of this study was to do a direct, within-group comparison of FPS and SCRs during fear conditioning in PTSD and trauma-exposed non-PTSD control groups, and relate each measure to specific PTSD symptoms. Although both measures have been used in previous PTSD research, they have not been compared and evaluated as potential biomarkers of symptoms. Given that PTSD is associated with dysregulated amygdala activity, it was hypothesized that FPS, but not skin conductance, would provide a better measure of group differences between PTSD and non-PTSD participants. Furthermore, based on our previous studies,^[12,^^30]^ it was hypothesized that FPS to the danger cue (CS+) and impaired inhibition of FPS in the presence of the safety cue (CS−) would be associated with PTSD symptom severity. As such, startle may provide a phenotype which is an optimal tool in translating basic science paradigms to clinical arenas.

## METHODS

### PARTICIPANTS

Participants were recruited as part of a larger study investigating the genetic and environmental factors that contribute to PTSD in a primarily African-American, low socioeconomic, inner-city population in Atlanta, GA.[Bibr b38][Bibr b39] All participants were recruited by study staff approaching them in the waiting rooms of primary care clinics at Grady Hospital. Exclusion criteria included active psychosis and major medical illnesses as assessed by history and physical examinations. Participants were also excluded for urine toxicology that was positive for cocaine or opiates and hearing impairment. Before their participation, all participants provided written informed consents approved by the Emory University Institutional Review Board. There were 111 participants included in the study, 70 traumatized individuals who did not meet criteria for PTSD (trauma controls), and 41 individuals who did (PTSD group).

### CLINICAL ASSESSMENT

The following measures were used to index PTSD symptoms, childhood trauma history and adult trauma history, respectively: PTSD Symptom Scale (PSS),^[40–42]^ Childhood Trauma Questionnaire (CTQ),^[43,^^44]^ and the Traumatic Events Inventory (TEI).^[42]^ These measures are based on self-report and have all been used previously in our work with this population and validated with structured clinical interviews.^[38]^

#### Modified PSS

This is a psychometrically valid 17-item self-report scale assessing PTSD symptoms over the two weeks before rating.^[40,^^42,^^45]^ The categorical definition of PTSD+ versus PTSD− was determined from responses to the DSM-IV-based PSS questionnaire A–E criteria (A, presence of trauma; B, presence of at least one re-experiencing symptom; C, presence of at least three avoidant/numbing symptoms; D, presence of at least two hyper-arousal symptoms; E, occurrence for at least 1 month). This instrument demonstrated high reliability with a Cronbach's α of .91 in the current sample. The average inter-item correlation was .40.

#### Childhood Trauma Questionnaire (CTQ)

The CTQ is a self-report inventory assessing childhood physical, sexual, and emotional abuse. Studies have established the internal consistency, stability over time, and criterion validity of both the original 70-item CTQ and the current brief version.^[43,^^45]^ The CTQ yields a total score and subscale scores for each of the types of child abuse. Cronbach's α for this sample was .85. The average inter-item correlation was .25.

#### Traumatic Events Interview

The TEI^[42]^ assesses lifetime history of trauma exposure and is a measure of both child abuse and adult trauma. The TEI assesses past experience and frequency of 13 separate types of traumatic events as well as feelings of terror, horror, and helplessness with such events.

### PSYCHOPHYSIOLOGICAL ASSESSMENT

The psychophysiological data was collected using Biopac MP150 for Windows (Biopac Systems, Inc., Aero Camino, CA). Electromyographic (EMG) and electrodermal activity (EDA) data were sampled at 1,000 Hz and amplified using the respective modules of the Biopac system. The acquired data were filtered, rectified, and smoothed in MindWare software (MindWare Technologies, Inc., Gahanna, OH) and exported for statistical analyses. EMG activity was recorded from two 5 mm Ag/AgCl electrodes placed over the *orbicularis oculi* muscle, approximately 1 cm under the pupil and 1 cm below the lateral canthus. The impedances for all participants were less than 6 kilo-ohms. The EMG signal was filtered with low- and high-frequency cutoffs at 28 and 500 Hz, respectively. Startle magnitude was assessed as the peak amplitude of the EMG contraction 20–200 ms following the acoustic stimulus. EDA was measured using two finger electrodes on the hypothenar surface of the nondominant hand, which served as ground electrodes for EMG. The SCR was analyzed for EDA, which was defined as the average increase (from a 1 s pre-CS onset baseline) from 3 to 6 s after the CS onset (each shape CS was presented on the computer monitor for 6 s).

### EXPERIMENTAL PROCEDURE

Participants were seated in a sound attenuated booth and asked to look at a computer monitor approximately 1 m in front of them. The startle probe (noise burst) was a 108 dB [A] SPL, 40 msec burst of broadband noise delivered binaurally through headphones. The fear conditioning protocol consisted of two phases: habituation and fear acquisition. The fear acquisition phase consisted of three blocks with four trials of each type (a reinforced conditioned stimulus, CS+; a nonreinforced conditioned stimulus, CS−; and the noise probe alone, NA). The habituation phase contained all the same trial types as the acquisition phase with the exception that none of the CSs were reinforced. Therefore, there was a total of 16 of each trial type (NA, CS+, CS−) during the session. The acquisition phase had a 100% reinforcement schedule, i.e., the CS+ was reinforced on every trial. Both CSs were colored shapes presented on a computer monitor for 6 s before the delivery of the startle probe, and co-terminated with the US.5 s after the presentation of the startle stimulus. The US was a 250 msec, 140-p.s.i airblast directed at the larynx as in our previous studies.^[31,^^46]^ In all phases, the inter-trial intervals were randomized to be 9–22 s.

### DATA ANALYSIS

Demographic and clinical data such as age, PTSD symptoms, and childhood and adult trauma history were compared between the PTSD+ and PTSD− groups using a one-way analysis of variance (ANOVA); categorical data, such as sex and race were analyzed using Chi square analyses.

FPS was defined as the change in the magnitude of the eyeblink response to the acoustic probe in the presence of the CS relative to the blink response to the acoustic probe in the absence of the CS. It was calculated using a Difference Score ([startle magnitude in the presence of a CS in each conditioning block]–[startle magnitude to the NA]). As noted above, SCR was calculated as the average response during the 3–6 s following CS onset minus the EDA baseline. The SCR data for each individual were square root transformed in order to normalize the data. Because we did not exclude low and nonresponders from the data set, the distributions showed slight positive skew (startle = 2.31, SCR = 1.68) and negative kurtosis (startle = 7.71, SCR = 3.57). These dependent variables were analyzed in a mixed ANOVA with the within-subject factor of Block (three levels for Acquisition), Trial Type (two levels, CS+ and CS−), and the between-groups factor of Diagnostic Group (two levels, PTSD+, control). Late Acquisition was defined as block 3 of Acquisition, when discrimination learning was at maximum; significant interactions were followed up by univariate analyses of covariance (ANCOVAs), with demographics, childhood trauma (CTQ), and adult trauma (TEI) used as covariates in all analyses involving diagnostic groups. The demographic variables were included as covariates in the between-group analyses because of reported sex differences in both startle measures^[47]^ and skin conductance measures;^[48,^^49]^ the trauma history variables were included in order to control for group differences in degree of trauma exposure. Stepwise regression analyses were conducted to examine the predictive value of the physiological measures to PTSD symptoms. Effect sizes for the analyses are shown as partial eta squared η^2^. All statistical analyses were performed in SPSS 17.0 for Windows (SPSS, Inc., Chicago, IL), with α = .05.

## RESULTS

### DEMOGRAPHIC AND CLINICAL DATA

There were no group differences in age, sex, or race (Table 1). However, participants with PTSD had higher levels of childhood trauma (CTQ score *F*(1, 108) = 14.58, *P*<.001) and adult trauma (TEI score *F*(1, 108) = 3.84, *P* = .05) than participants who did not meet criteria for PTSD. As expected, PTSD participants had higher total PTSD symptoms scores (*F*(1, 108) = 160.42, *P*<.001) and higher symptom cluster sub-scores for re-experiencing (*F*(1, 108) = 78.70, *P*<.001), and hyper-arousal (*F*(1, 108) = 94.11, *P*<.001) compared to the control group.

**Table 1:** Demographic and clinical data for the study sample

Demographics	PTSD (*n* = 41)	Control (*n* = 70)	
Sex (% women)	58.5	57.1	ns
Race (% AA)	95.1	94.3	ns
Age (*M*, *SD*)	41.15 (12.03)	40.50 (12.31)	ns
Trauma history			
Childhood trauma (*M*, *SD*)	51.93 (19.34)	39.24 (15.00)	*P*<.0001
Adult trauma (*M*, *SD*)	3.12 (2.11)	2.36 (1.87)	*P* = .05
PTSD symptoms			
Total (*M*, *SD*)	27.23 (10.12)	7.36 (6.20)	*P*<.0001
Re-experiencing (*M*, *SD*)	6.67 (3.99)	1.49 (2.06)	*P*<.0001
Avoidance (*M*, *SD*)	11.24 (4.62)	2.87 (3.56)	*P*<.0001
Hyper-arousal (*M*, *SD*)	9.29 (3.66)	3.00 (3.01)	*P*<.0001

### FEAR-POTENTIATED STARTLE

A three-way ANOVA examining the effects of Block (three levels) by Trial Type (two levels) between Diagnostic Group (PTSD, control) on startle revealed a main effect of Block, *F*(2, 218) = 17.37, *P*<.001, η^2^ = .14, and Trial Type, *F*(1, 109) = 11.42, *P* = .001, η^2^ = .10. There was also an interaction of Block and Trial Type, *F*(2, 218) = 8.41, *P*<.001, η^2^ = .07, with FPS discrimination between CS+ and CS− increasing over Blocks. Finally, there was a main effect of Group, *F*(1, 109) = 5.02, *P*<.05, η^2^ = .04, with PTSD participants displaying greater levels of FPS overall; there were no significant interactions between Group and Trial Type or Group and Block.

**Figure 1:**
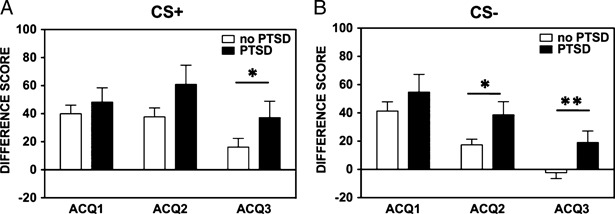
Fear-potentiated startle between PTSD and controls by fear acquisition block, separated by trial type. (A) shows group differences in fear-potentiated startle across blocks during the CS+; (B) shows the group differences during the CS−. The value on the *Y* axis depicts difference score calculated as startle magnitude to each CS minus startle magnitude to noise probe alone.

The significant interaction was followed by a comparison of Trial Types within each Block. There were no significant differences during Block 1. However, in Blocks 2 and 3, startle responses to CS+ were significantly greater than startle to CS−, *F*(1, 109) = 14.85, *P*<.001, η^2^ = .12, and *F*(1, 109) = 13.52, *P*<.001, η^2^ = .11, respectively. Given the a priori hypothesis that there would be specific group differences for CS+and CS− using startle measures, we performed univariate ANCOVAs comparing PTSD participants to controls within each trial type. Between-group comparisons of the startle data for Blocks 2 and 3 using ANCOVAs with age, sex, race, childhood and adult trauma levels as covariates, showed that PTSD participants had reduced inhibition of FPS compared to controls to the CS− in Block 2, *F*(1, 101) = 6.13, *P*<.05, η^2^ = .06, and Block 3, *F*(1, 101) = 9.82, *P*<.01, η^2^ = .09, and increased FPS to the CS+in Block 3, *F*(1, 101) = 6.13, *P*<.05, η^2^ = .06. Figure 1 shows FPS responses across groups.

### SKIN CONDUCTANCE RESPONSE

**Figure 2:**
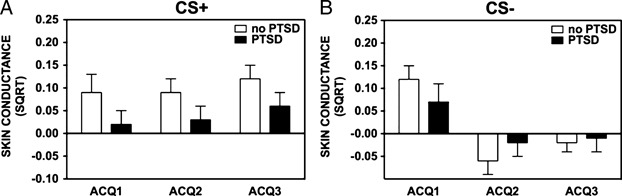
Skin conductance response between PTSD and controls by fear acquisition block, separated by trial type. (A) shows group differences in fear-potentiated startle across blocks during the CS+; (B) shows the group differences during the CS−. The value on the *Y* axis depict square root transformations of the SCR calculated at the level of electrodermal activity during the 3–6 s post-CS onset minus the electrodermal activity during the 1 s before CS onset.

The same variables were used in a three-way ANOVA examining these effects on SCR. As was the case with startle, results show a main effect of Block, *F*(2, 218) = 6.43, *P*<.01, η^2^ = .06, and Trial Type, *F*(1, 109) = 10.91, *P* = .001, η^2^ = .09, as well as an interaction of Block and Trial Type, *F*(2, 218) = 14.48, *P*<.001, η^2^ = .12, with discrimination between the CS+and CS− developing over the three Blocks. Unlike startle, there was not a significant effect of Group, *F*(1, 109) = 0.8, *P*>.1, η^2^ = .01. Again, the significant interaction was followed up by a comparison of Trial Types within each Block. In Block 1 there was a trend for a difference between trials, *F*(1, 109) = 3.29, *P* = .07, η^2^ = .03; in Blocks 2 and 3, the CS+response was significantly greater than the CS−, *F*(1, 109) = 16.63, *P*<.001, η^2^ = .13, and *F*(1, 109) = 16.21, *P*<.001, η^2^ = .13, respectively. Figure 2 shows SCR between groups.

In order to correlate the two measures, the average FPS and SCR values for each Trial Type was calculated, as well as a differential conditioning score by subtracting the value of CS− from that of CS+for both measures. Although the absolute FPS and skin conductance measures were not significantly correlated, the differential score was positively correlated between FPS and SCR, *r*(111) = .22, *P*<.05. We also examined the correlations between the two psychophysiological measures and PTSD symptoms within each of the three clusters: re-experiencing, avoidance, and hyper-arousal. Potentiated startle responses were positively correlated with re-experiencing and hyper-arousal symptoms, whereas SCR was negatively correlated with hyper-arousal symptoms (Table 2).

**Table 2:** Correlations between psychophysiological measures and PTSD symptom clusters

	Startle to danger cue	SCR to danger cue	Startle to safety cue	SCR to safety cue
Re-experiencing PTSD symptoms	.24^*^^*^	−.05	.22^*^	.06
Avoidance PTSD symptoms	.05	−.12	.12	.04
Hyper-arousal PTSD symptoms	.14	−.22^*^	.23^*^	−.04

### REGRESSION ANALYSES

Our previous studies indicated that startle responses to danger cues (CS+) predicted re-experiencing symptoms, whereas responses to safety cues (CS−) predicted hyper-arousal symptoms of PTSD. In order to examine whether FPS or SCR independently contribute to variance in PTSD symptoms, two regression analyses were performed, one with re-experiencing symptoms (Table 3A) and one with hyper-arousal symptoms (Table 3B). In the first regression, a stepwise method was used in which demographic data was entered in step one, trauma history was entered in step two, average FPS to the CS+ was entered in step three, and average SCRs to the CS+ were entered in the final step. The overall model with all four predictors was significant, *F*(7, 105) = 3.27, *P*<.01) and accounted for 18.9% of the variance in re-experiencing symptoms. After accounting for demographics and trauma history, startle alone accounted for 5.6% of the variance, *F*_change_(1, 99) = 6.76, *P* = .01. SCR did not significantly contribute to re-experiencing symptoms, *F*_change_(1, 99) = 0.39, *P*>.1. The regression analysis was repeated with SCR added in the 3rd step and startle added in the last step, but the significance of the results did not change (see Table 3A for respective β values).

**Table 3:** Stepwise regression analysis of (A) re-experiencing symptoms, and (B) hyper-arousal symptoms of PTSD

(A) Outcome: Re-Experiencing PTSD symptoms	*R*^2^	*R*^2^ change	*F* change	*P*
Predictors				
1. Age, sex, and race	.010	.014	0.50	ns
2. Childhood and adult trauma	.130	.116	6.67	.002^*^^*^^*^
3. Startle to danger cue	.186	.056	6.76	.01^*^^*^
4. SCR to danger cue	.189	.003	0.39	ns
Final model: *F*(7, 105) = 3.27, *P* = .004	β	Part *r*	*P*	
Age	.01	.01	ns	
Sex	−.04	−.04	ns	
Race	.06	.05	ns	
Childhood trauma	.38	.35	<.001	
Adult trauma	−.05	−.04	ns	
Startle to danger cue	.26	.24	.01	
SCR to danger cue	−.06	−.06	ns	
(B) Outcome: Hyper-arousal PTSD symptoms	*R*^2^	*R*^2^ change	*F* change	*P*
Predictors				
1. Age, sex, and race	.003	.003	0.11	ns
2. Childhood and adult trauma	.134	.131	7.56	.001^*^^*^^*^
3. Startle to safety cue	.191	.057	7.00	.009^*^^*^
4. SCR to safety cue	.194	.003	0.32	ns
Final model: *F*(7, 105) = 3.37, *P* = .003	β	part *r*	*P*	
Age	.03	.03	ns	
Sex	−.17	−.15	ns	
Race	.07	.07	ns	
Childhood trauma	.39	.36	<.001	
Adult trauma	−.09	−.08	ns	
Startle to safety cue	.25	.24	.01	
SCR to safety cue	−.06	−.05	ns	

In a separate stepwise regression predicting hyper-arousal symptoms (Table 3B) with demographics and trauma history in the first two steps as above, FPS to the CS− in step three, and SCR to CS− in the final step accounted for 19.4% of the variance in symptoms, *F*(7, 105) = 3.37, *P*<.01. As above, after accounting for demographics and trauma history, FPS to safety cues significantly predicted hyper-arousal, *F*_change_(1, 99) = 7.00, *P*<.01. SCR to safety cues did not significantly contribute to hyper-arousal symptoms, *F*_change_(1, 99) = 0.32, *P*>0.1. Again, the same regression analysis was conducted by reversing the step order for SCR and startle, but only startle had a significant association with hyper-arousal symptoms (see β values in Table 3B).

## DISCUSSION

PTSD is characterized by excessive fear responding to trauma reminders that persist in the presence of safety reminders. Human neuroimaging studies suggest that this is, in part, due to the interplay of overactive, amygdala-driven excitatory circuits and deficient, higher-order inhibitory circuits.^[17,^^50]^ The objective of this study was to examine the psychophysiology of fear acquisition in a highly traumatized civilian population in order to shed light on the neural underpinnings of maladaptive fear in PTSD. Furthermore, the study aimed to compare FPS and SCRs during fear conditioning in their respective associations with specific PTSD symptom clusters.

A translational differential fear conditioning model was used to measure conditioned fear in PTSD and trauma-exposed control participants during both aversive (CS+) and safety (CS−) conditions. To address a discrepancy in findings from researchers that measured FPS versus SCR to assess fear discrimination in PTSD, these two fear measures were directly compared under the same experimental conditions. Based on previous findings and the differing neurobiology of these two fear measures,^[18]^ it was predicted that FPS, but not SCR, would show group differences between PTSD and control participants.

Results showed that all participants displayed robust fear expression to the danger cue and significant discrimination between the danger and safety cue, which was captured in both measures, indicating successful fear conditioning. Furthermore, participants with PTSD expressed significantly greater fear to both the danger and safety cues than the trauma controls. This heightened fear was only captured with FPS, whereas SCR did not differ between groups. These findings replicate earlier studies showing exaggerated FPS to danger cues in PTSD subjects^[30]^ and the validity of FPS as a measure of fear conditioning in human populations.^[32,^^51]^ They also replicate studies showing SCR to be a reliable measure of differential fear conditioning.^[16,^^21,^^34]^ Nevertheless, the current findings suggest that SCR may not be as sensitive a measure as FPS to diagnostic differences in fear expression.

To further explore this idea, independent contributions of FPS and SCR to PTSD variance were examined using regression analyses of re-experiencing symptoms and hyper-arousal symptoms measured from the PSS. Our previous studies demonstrated that FPS responses to danger cues (CS+) predicted re-experiencing symptoms,^[32]^, ^[52]^ whereas fear inhibition to safety cues predicted hyper-arousal symptoms of PTSD.^[31]^ This may seem unexpected at first glance, given that exaggerated startle is indicated as part of the hyper-arousal cluster in DSM-IV. However, hyper-arousal in PTSD is also related to hyper-vigilance, irritability and difficulties in sleeping and concentration,^[1]^ which speaks more to a lack of inhibitory control in general, and perhaps, more specifically, an inability to inhibit fear in safe situations. The re-experiencing cluster may be more related to a hyperactive amygdala response to trauma reminders,^[13]^ which, in this study, is manifested as heightened FPS to CS+ (i.e., danger cue) in PTSD.

This study found a weak positive correlation between startle and SCR measures of fear. Although SCR is very frequently used as the sole measure of autonomic nervous system conditioning,^[19]^ multiple physiological measures may be necessary for assessment of fear in clinical populations. Although previous research has used several psychophysiological measures to differentiate among anxiety disorder patients,^[53,^^54]^ only a small number of studies have examined these biomarkers along the symptom spectrum within symptom clusters. This study replicated our earlier findings showing that startle to danger cues significantly accounted for the variance in PTSD re-experiencing symptoms.^[32]^ Unlike startle, SCR did not significantly contribute to re-experiencing symptoms. Similarly, impaired inhibition of FPS to safety cues significantly predicted hyper-arousal, whereas SCR to safety cues did not. It is important to note that several studies have found that decreased SCR habituation may be associated or even predictive of PTSD.^[55,^^56]^ It has been suggested that SCR is closely tied to contingency awareness that may involve activation of the hippocampus,^[57]^ a structure that may be linked to vulnerability for PTSD.^[58]^ On the other hand, the startle reflex is distinct from SCR in that it is directly modulated by the amygdala,^[18]^ and associated with valence rather than arousal and novelty.^[37]^ A recent study found that propranolol interrupted reconsolidation of fear memory when measured with FPS, but not SCR,^[59]^ further suggesting the specificity of startle as a measure of amygdala dependent, cue-specific fear.

When using psychophysiological methods as translational tools to understand PTSD and develop objective measures that track treatment outcome, it may prove more effective to use methods that translate brain activity of the areas known to be involved in the neurobiology of PTSD symptoms. As such, FPS seems to provide an ideal translational tool for investigating the neurobiological underpinnings of PTSD symptom exacerbation, such as amygdala dysregulation, whereas SCR may not be as sensitive to these alterations. Another advantage of FPS is the availability of animal models, such as rodents^[26,^^27]^ and nonhuman primates,^[28]^ which allow for basic investigation of neural underpinnings of fear expression and fear inhibition that can be used in development of fear inhibition-based therapeutic approaches for PTSD, such as exposure therapy.^[60]^

The nature of this study sample provides both strengths and limitations. One limitation is the use of a self-report measure (PSS) to determine PTSD diagnosis. However, the PSS has good psychometric qualities, and strong validity with clinician-administered measures, such as the CAPS,^[61]^ and has been well validated in our population.^[38,^^39,^^42]^ A strength of this study is statistically controlling for degree of trauma exposure between the PTSD and control participants. Although other studies have also used trauma-exposed control groups,^[35]^ there appears to be a linear “dose response” association between trauma load and PTSD symptoms,^[38]^ such that even trauma exposed individuals without PTSD may have significantly lower levels of trauma than individuals who meet DSM criteria for PTSD. Interestingly, the regression analyses indicate that heightened FPS is significantly related to PTSD symptoms beyond those accounted for by the trauma, thus underscoring its potential use as an objective measure of symptom severity. Finally, participants in this study were recruited from the primary care patient pool at Grady Hospital in Atlanta, which serves mainly an African-American, low-socioeconomic status, highly traumatized population. This population is significantly more susceptible to trauma-related stress disorders^[39]^ and has been largely understudied in human clinical research. This study represents a much-needed effort toward understanding the psychological outcomes of similarly vulnerable populations.

In summary, we found that FPS provided a sensitive measure of heightened fear responses in PTSD, which were associated with specific clinical symptom presentations. Furthermore, the ability to translate FPS paradigms across species allows us to use genetics, biochemical and pharmacological research techniques. Given that the groundwork in fear neurocircuitry has been greatly developed in animal models, the field of PTSD research is well poised to discover novel approaches for prevention, intervention, and treatment of the disorder.
